# Comparative content analysis and biological activity studies of fatty and essential oils from some garlic products sold in Turkish community pharmacies, natural, and fermented garlic

**DOI:** 10.1002/fsn3.4352

**Published:** 2024-07-21

**Authors:** Elif Ulutaş Deniz, Hafize Yuca, Bilge Aydın, Gözde Öztürk, Furkan Çoban, Gamze Göger, Betül Demirci, Songül Karakaya

**Affiliations:** ^1^ Department of Pharmacy Management, Faculty of Pharmacy Atatürk University Erzurum Turkey; ^2^ Department of Pharmacognosy, Faculty of Pharmacy Atatürk University Erzurum Turkey; ^3^ Department of Pharmacognosy, Faculty of Pharmacy Erzincan Binali Yıldırım University Erzincan Turkey; ^4^ Department of Pharmacognosy, Faculty of Pharmacy Anadolu University Eskişehir Turkey; ^5^ Department of Field Crops, Faculty of Agriculture Ataturk University Erzurum Turkey; ^6^ Department of Pharmacognosy, Faculty of Pharmacy Afyokarahisar Health Sciences University Afyon Turkey; ^7^ Department of Pharmaceutical Botany, Faculty of Pharmacy Atatürk University Erzurum Turkey

**Keywords:** anticholinesterase, antidiabetic, antimicrobial, antioxidant, fermented, garlic

## Abstract

Garlic (Alliaceae), an annual herb, is renowned not only for its distinctive flavor but also for its extensive therapeutic applications in managing various ailments and health conditions. In this study, garlic products identified as the best‐selling items in Turkish pharmacies for various purposes were compared with garlic grown under standard conditions in terms of chemical composition and antidiabetic, anticholinesterase, antimicrobial, and antioxidant properties. Three of garlic samples were prepared by researchers. According to survey results, the most commonly sold garlic‐related products in pharmacies are black garlic extract tablets (4), capsules (5), garlic oil (6), garlic oil pearls (7), and fermented garlic (8). Diallyl disulfide (DADS) was identified as a predominant compound in sampled oils, ranging from 4.9% to 48.6%. Another noteworthy finding is the identification of allyl methyl disulfide and allyl methyl trisulfide as major components in sampled oils, with concentrations spanning from 2.9% to 9.8% and 0.4% to 17.5%, respectively. In both 2,2′‐azino‐bis‐(3‐ethylbenzothiazoline‐6‐sulfonic) acid (ABTS^·+^) and 2,2‐diphenyl‐1‐picrylhydrazyl (DPPH^•^) tests, Sample 1 (fermented) exhibited the highest antioxidant activity. Sample 3 (cultivated) was richer in total phenol–total tannin content. Sample 6 exhibited the highest α‐glucosidase inhibition among antidiabetic activities, reaching 28.93%. Sample 5 capsules demonstrated the highest *α*‐amylase inhibition at 51.50%. Sample 7 exhibited the most notable inhibition against both acetylcholinesterase (22.92%) and butyrylcholinesterase (13.37%). Samples 3, 6, and 8 were found to be more effective against *Candida tropicalis* with minimum inhibitory concentration (MIC) = 625 μg/mL. A comprehensive study on garlic products, including popular items from Turkish pharmacies and those grown under standard conditions, revealed diverse chemical compositions and multifaceted health properties.

## Graphical abstract

Antidiabetic activity of Sold and Natural Garlic Fatty and Essential Oils. Anticholinesterase activity of Sold and Natural Garlic Fatty and Essential Oils. Antioxidant activity of Sold and Natural Garlic Fatty and Essential Oils. Antimicrobial activity of Sold and Natural Garlic Fatty and Essential Oils. Diallyl disulfide (DADS) was identified as a predominant compound in the sampled oils.

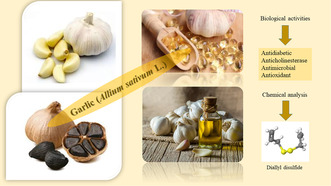

## 1 INTRODUCTION

Recent findings of garlic from the Late Bronze Age site of settelement known as Akrotiri on the Greek Island of Thera have sparked research examining its historical, economic, and social implications in the archeological narrative. Garlic holds a significant place in our culture, medicine, mythology, culinary practices, and ethnography, and has become an integral part of our daily lives. While we previously recognized its importance in historical periods, particularly since Roman times, when its use spread throughout Europe and beyond, archeobotanical investigations have expanded our understanding of this crop in both prehistoric and historical contexts. These findings shed light on how garlic was perceived, its potential uses, and its evolving significance over time (Sarpaki, 2021). Garlic (*Allium sativum* L.) has perpetually occupied a central position, straddling the realms of culinary delights and medicinal plants throughout the annals of history. Few items can rival the pervasive use of garlic, which stands as one of the earliest known plants employed to enhance flavor in cuisines across numerous civilizations globally. Its ubiquitous presence in the culinary world serves as a testament to its enduring popularity. Moreover, garlic epitomizes the quintessence of healthy foods, a notion that dates back to over 2300 years, echoing the wisdom of Hippocrates, who advocated for the medicinal potential of food with the famous words, “Let your food be your medicine…”. In Western and Central European languages, the terminology for onion can be traced back to Latin roots. Unlike garlic, which consists of multiple cloves beneath its outer covering, the onion is spherical and singular in structure. This distinction likely influenced its nomenclature, evolving from “unio” to “union” and eventually “oniones,” leading to the modern term “onion.” On the other hand, the linguistic roots for garlic can be identified in ancient Rome as “allium” or “alium,” reflected in its names in Italian (“aglio”), Spanish (“ajo”), and French (“ail”). Haller and Linnaeus, in the 18th century, adopted the ancient Roman name as the scientific nomenclature for garlic (Ekşi et al., 2020).

In Greek mythology, Apollo's lover Coronis, who was pregnant with his child, took the opportunity to be with her beloved Ischys while Apollo was away in Delphi. Apollo had left a white‐feathered crow as a watcher with Coronis when he went to Delphi. However, Coronis managed to carry out her plan by deceiving the crow. The bad news reached Delphi soon after. Apollo, consumed by anger, first cursed the crow for failing to watch over his lover and turned its white feathers into black; thus, the entire crow species was condemned to darken. He then killed Ischys with his arrows. Apollo's sister, the goddess Artemis, dealt with Coroni’ punishment by riddling her with arrows. Apollo was deeply troubled, experiencing the pain of both the betrayal he endured and the loss of the woman he loved. To alleviate his pain and ease his conscience, he sought help from Hermes. Hermes, by tearing apart Coronis's body engulfed in flames, extracted the still‐living baby. This child was named Asclepius. Apollo entrusted his son to the centaur Chiron. Chiron taught Asclepius the art of healing. Consequently, Asclepius became a skilled healer while still a young man. When Athena gifted him with the magical blood of Medusa, Asclepius gained the ability to resurrect the dead in addition to his healing skills. His reputation soared, especially due to his ability to bring the dead back to life, disturbing Zeus. The resurrection of the dead and the use of magical remedies could disrupt the order of the world. Zeus believed Asclepius had gone too far and struck him with lightning. As Asclepius fell, he held the recipe for immortality in his hand. As his body touched the earth, the recipe mixed with the soil. The secret of immortality blended with the raindrops falling from the sky and merged with the earth, giving rise to a brand‐new plant. This plant, said to cure a thousand ailments, was garlic. In Romani mythology, there are disease spirits that plague people. One of the most famous of these spirits, Melalo (the dirty one), advises his father to give a person garlic. Following Melalo's advice, the man urinates on the garlic and feeds it to his wife. The woman who consumes the garlic becomes pregnant and gives birth to Bitoso (the fasting one), a worm. Bitoso causes people to experience stomachaches and headaches, leading to coughs and loss of appetite. Like its sibling onion, garlic was believed to be protective against evil spirits. Therefore, garlic was hung in various parts of the house. In myths, garlic is used as a protective charm against supernatural beings, much like vampires (Gezgin, 2007).

Garlic, an annual herb from the Alliaceae family, is renowned not only for its distinctive flavor but also for its extensive therapeutic applications in managing various ailments and health conditions. Its historical usage spans culinary, medicinal, and even spiritual domains. Fresh garlic is a rich source of vitamins, minerals, trace elements, and essential oils that contribute to overall human health. The potent chemical constituents in garlic underpin its diverse pharmacological activities, including antimicrobial, anti‐inflammatory, anticancer, cardiovascular, and immunomodulatory properties (Saif et al., 2020).

Garlic, celebrated for its robust aroma attributed to organosulfur compounds like allicin, is widely recognized as a staple culinary seasoning. Beyond its culinary applications, garlic holds significant promise in ethnomedicine for addressing an array of health conditions. These encompass hypertension, pneumonia, hair loss, snakebite, diabetes, wounds, cough, paralysis, scabies, malaria, hemorrhoids, carbuncles, heart diseases, asthma, pain, respiratory disorders, influenza, and female infertility. These therapeutic advantages can be primarily attributed to garlic's diverse properties, including antidiabetic, antiatherosclerotic, antimicrobial, antihypertensive, anticancer, cardioprotective, diuretic, aphrodisiac, sedative, carminative, and antipyretic effects. A multitude of studies substantiate these properties, establishing garlic as a potent natural remedy with extensive potential beyond its traditional culinary role (Tudu et al., 2022).

In a time predating antibiotics and modern pharmaceuticals, a single bulb of garlic encompassed an entire pharmaceutical industry within itself due to its extensive range of effects. Various suppositions about this herb surfaced; while some proved futile and faded away, others have persevered through the ages. Garlic earned various names that persist today, including “Russian penicillin,” “natural antibiotic,” “vegetable viagra,” “plant talisman,” “rustic's theriac,” and “snake grass.” The evolving ideas and understanding associated with garlic have empowered pharmacists and physicians, enabling them to effectively address the challenges within their profession and enhance the quality of human life. Garlic remains an indispensable plant in daily life, transcending through time from ancient civilizations to the contemporary era. Packed with active compounds, garlic exerts its effects on nearly every aspect of the human body. It stands as an exceptional tonic for the human organism and has been utilized for medicinal purposes throughout history (Petrovska & Cekovska, 2010).

Garlic is a food ingredient widely used in our gastronomy. What are the main compounds that give freshly cut *Allium* species their distinctive flavor? This question holds significant relevance for the food and flavor industry as well as for chemotaxonomic studies. Cultivated garlics are characterized by high levels of S‐allyl compounds and low levels of S‐methyl, S‐propyl, and S‐propenyl compounds. In contrast, the wild *Allium* species analyzed exhibited all four moieties, with variations observed both between different species and among different organs of the same plant (Block et al., 1992; Boscher et al., 1995; Corzo‐Martinez et al., 2007).

This study investigates the best‐selling garlic products in Turkish pharmacies, comparing them with three distinct samples: a fermented variant (Sample 1), one subjected to a 30‐day drying process at 60°C in an incubator (Sample 2), and a directly cultivated sample (Sample 3). The comparison focuses on chemical composition and evaluates properties related to antidiabetic, anticholinesterase, antimicrobial, and antioxidant activities. By examining these parameters, the study aims to provide a comprehensive understanding of the differences and potential health benefits associated with each preparation method.

## 2 MATERIALS AND METHODS

### 2.1 Determination of garlic products commonly consumed in community pharmacies

A cross‐sectional, descriptive study was conducted between September and November 2023 among pharmacists registered with the Erzurum Chamber of Pharmacists and currently practicing pharmacy in order to determine the garlic products frequently sold in community pharmacies. A questionnaire was distributed to community pharmacists registered with the Erzurum Chamber of Pharmacists via WhatsApp. The questionnaire outlined the participation criteria and elucidated the significance of the study. The survey remained open for approximately 2 months, with reminders sent every 10 days to encourage participation. In total, 35 respondents successfully completed the survey. Two instruments were employed to gather the required data: a sociodemographic data form and the *form on the sales practice of garlic products*. These two scales were integrated into a single questionnaire and administered through Google Forms. A researcher‐developed form was employed to collect descriptive information about the pharmacists. This *sociodemographic data form* comprised seven questions aimed at gathering details on the pharmacists’ gender, age, years of experience in the profession, educational level, and pharmacy location. *Form on the sales practice of garlic products*, a questionnaire developed by the researchers, drawing upon the existing literature, encompasses inquiries about pharmacists’ sales practices, covering aspects such as the sale of herbal products, specifically garlic products. Questions delve into the types of garlic products sold and the primary purposes for which these products are most frequently sold. Descriptive statistics, such as frequency and percentage, were used to describe the distribution of study variables.

### 2.2 Plant material

The garlic (*Allium sativum* L.) sample used in the study was obtained from local farmer fields in Alatarla Village (41°29′21″°N, 34°01′58″°E), 624 m above sea level of Taşköprü district of Kastamonu province in Turkey, where garlic is intensively cultivated. The garlic material is a product in the European Union (EU) Geographical Indication Products List called softneck garlic. Fifteen plants were sampled randomly from fields. Here are the physical characteristics of the material used: The headshell color is off‐white, with a bulb weight of 23.85 g. There are 14.8 cloves on the head, consisting of 5.3 large cloves and 9.5 small cloves. The large cloves weigh 2.72 g each, while the small cloves weigh 1.68 g each. Harvested on July 10, 2023, the garlic underwent a shade‐drying procedure for subsequent analysis, with storage maintained at +4°C.

### 2.3 Acquisition of garlic samples and pharmaceutical products

In the study, a total of eight samples were used. Three of these samples were prepared by the researchers. One was fermented (1), one was dried at 60°C for 30 days in an incubator (2), and one was a directly cultivated sample (3). For the fermented samples, again 50 g was weighed, wrapped with cling film, and sealed with aluminum foil. It was then kept in the same incubator for 30 days at a temperature of 60°C and a relative humidity of 70%–80%. For the dried sample, 50 g of garlic was weighed, and it was dried in the incubator at 60°C for 30 days.

According to the survey results, the most commonly sold garlic‐related products in pharmacies are black garlic extract tablets (4) (each tablet contains 750 mg of garlic extract), capsules (5) (each capsule contains 300 mg of garlic powder plus excipients), garlic oil (6) (it has been obtained by cold‐pressing), garlic oil pearls (7) (garlic oil concentrate of 1 mg (*Allium sativum*) from approximately 500 mg of fresh garlic bulbs), and fermented garlic (8) (Taşköprü garlic). Therefore, these five products were obtained from pharmacies.

### 2.4 Solid‐phase microextraction (SPME) method

The solid‐phase microextraction (SPME) method was employed for the analysis of volatile compounds in the garlic samples mentioned above. This technique relies on the adsorption of volatile compounds onto a fiber coated with a polymeric stationary phase, followed by thermal desorption of the retained compounds at the injection port of a gas chromatograph (GC). For the analysis, 2 g of the sample was placed in a vial, and SPME was conducted at 50°C for 15 min. Subsequently, gas chromatography (GC) analysis was performed to identify and quantify the volatile compounds.

### 2.5 Headspace–SPME

The manual SPME device (Supelco, Bellefonte, PA, USA) with a fiber precoated on a 65‐μm thick layer of polydimethylsiloxane/divinylbenzene (PDMS/DVB‐blue) was used for extraction of the garlic volatiles. The vial containing the garlic sample (2 g) was sealed with parafilm. The fiber was pushed through the film layer for exposure to the headspace of the extract for 15 min at 50°C. The fiber was then inserted immediately into the injection port of the gas chromatograph–mass spectrometer (GC–MS) for the desorption of the adsorbed volatile compounds for analysis.

#### 2.5.1 Analysis of volatile compounds

The volatiles were analyzed by GC–MS using a Hewlett Packard GCD system. An HP‐Innowax FSC column (60 m × 0.25 mm inner diameter, with 0.25 mm film thickness) was used with helium as carrier gas (1 mL/min). The GC oven temperature was kept at 60°C for 10 min and programmed to 220°C at a rate of 4°C/min, then kept constant at 220°C for 10 min, and then programmed to 240°C at a rate of 1°C/min, at splitless mode. The injector temperature was kept at 250°C. Electron ionization (EI)‐mass spectra were recorded at 70 eV. The mass range varied from 35 to 425 m/z.

#### 2.5.2 Identification of the components

Identification of the components was carried out by comparison of their relative retention times with those of authentic samples or by comparison of their relative retention index (RRI) to series of *n*‐alkanes. Computer matching against commercial (McLafferty & Stauffer, 1989) and in‐house “Başer Library of Essential Oil Constituents” was built up by genuine compounds and components of known oils (Hochmuth & Sparkman, 2007).

#### 2.5.3 Methylation – obtaining fatty acids

As much as 0.2 g of the sample was weighed and transferred to a 250 mL flask. Subsequently, 5 mL of 0.5 N methanolic sodium hydroxide (NaOH) was added. The mixture was refluxed and heated in a cooling condenser for 10 min. Afterward, 5 mL of boron trifluoride in methanol solution (BF3/MeOH) was added, and the heating was continued for additional 2 min. Following this, 5 mL of hexane was added, and the mixture was allowed to cool. Once cooled, the contents were transferred to a 25 mL separatory funnel and made up to 250 mL with saturated salt solution. The upper phase was separated using a separatory funnel and subjected to gas chromatography–flame ionization detection (GC–FID) and GC–MS analyses.

#### 2.5.4 Preparation of fatty acid methyl esters

As much as 0.2 g of the sample was refluxed with 5 mL of 0.5 N sodium hydroxide solution in methanol for 10 min. Then, 5 mL of 14%–20% BF3 in methanol solution was added through the condenser and boiled for further 2 min. Five milliliters of *n*‐hexane was added and boiled for a further 1 min. The solution was cooled and 5 mL of saturated sodium chloride (NaCl) solution was added and the flask was rotated very gently several times. Additional saturated NaCl solution was added to float the hexane solution into the neck of a 1 mL flask and the upper hexane solution was transferred into a vial and then analyzed by GC‐FID and GC–MS systems, simultaneously.

### 2.6 Antidiabetic activity

#### 2.6.1 α‐Glucosidase inhibition assay

The impact on *α*‐glucosidase inhibition was evaluated using a modified method adapted from Bachhawat et al. (2011), as outlined by Yuca et al., (2021). The samples and acarbose were dissolved in a potassium phosphate (KH_2_PO_4_) buffer (50 mM, pH 6.9). In a 96‐well plate, 20 μL of the samples, 10 μL of the enzyme solution (1 U/mL), and 50 μL of the potassium phosphate buffer were combined and incubated at 37°C for 5 min. The reaction was initiated by adding 20 μL of *p*‐nitrophenyl‐*α*‐D‐glucopyranoside (3 mM), and the mixture was further incubated for 30 min at 37°C. Termination of the reaction involved adding 50 μL of 0.1 M sodium carbonate (Na_2_CO_3_). Acarbose was used as the positive control. The resulting *p*‐nitrophenol was measured at 405 nm using a microplate reader. Each experiment was replicated three times.

The percentage inhibition was calculated as (1 − ΔA_405sample_/ΔA_405control_) × 100.

#### 2.6.2 α‐Amylase inhibition assay

The inhibitory impact on *α*‐amylase was determined using a modified version of the method outlined by Nampoothiri et al. (2011), as adapted by Yuca et al. (2021). The samples and acarbose were dissolved in dimethyl sulfoxide (DMSO). The samples (100 μL) were blended with a 1% starch solution (100 μL) in a 20 mM sodium phosphate buffer (pH 6.9 with 6 mM sodium chloride) and incubated at 25°C for 10 min. Subsequently, upon the addition of *α*‐amylase solution (0.5 mg/mL), further incubation was carried out for additional 10 min. The reaction was terminated with a dinitrosalicylic acid color reagent, and the absorbance at 540 nm was recorded. Acarbose was employed as the positive control. Each experiment was replicated three times.

The percentage inhibition was calculated as (1 − ΔA_540sample_/ΔA_540control_) × 100.

### 2.7 Anticholinesterase activity

#### 2.7.1 Acetylcholinesterase (AChE) and Butyrylcholinesterase (BChE) inhibition assay

The inhibitory activities against AChE and BChE were assessed utilizing the methodology outlined by Ingkaninan et al. (2000) with minor adjustments. The samples and donepezil were dissolved in DMSO. In a 96‐well plate, a mixture of 5,5′‐dithiobis 2‐nitrobenzoic acid (DTNB, Ellman's reagent), substrate (acetylthiocholine for AChE; butyrylthiocholine for BChE), Tris–HCl buffer, and samples was subjected to incubation, followed by the addition of either AChE or BChE. The inhibition was determined through spectrophotometric measurement at 405 nm, with donepezil serving as the positive control. Each experiment was replicated three times.

The percentage inhibition was calculated as (1 − Δ_A405sample_/ΔA_405control_) × 100.

### 2.8 Antioxidant and free radical scavenging activity

In antioxidant activity tests, first, the activities of the standards in a wide concentration range were evaluated in terms of percentage inhibition. In both experiments, the concentration that could be significant and at which the standards reached maximum percentage inhibition was determined. Dosages that could be utilized within this concentration range were identified for our extracts. The most meaningful dose to which our extracts and standards can be compared has been determined as 50 μg/mL. Since the antioxidant capacities of the extracts are lower compared to the standards, the IC_50_ (half‐maximal inhibitory concentration) value was not calculated, and the results are expressed in terms of percentage (%) inhibition to reveal meaningful data. It is possible to encounter results expressed in percentage (%) inhibition when reviewing the literature.

#### 2.8.1 ABTS ^·+^ scavenging activity

The ABTS cation radical scavenging activity was determined according to the study by Re et al. (1999) 2 mM ABTS^•+^ solution was used as free radical, while *α*‐tocopherol (TK) and trolox (TR) were used as standard antioxidants. Stock solutions were prepared at 10 different concentrations (5–100 μg/mL) of the extracts and standards. The absorbances of whole samples were read at 734 nm against the blank consisting of phosphate buffer. All measurements were repeated three times to ensure accuracy and precision in the study.

The ABTS^•+^ radical scavenging capacity was calculated according to the following equation:

$$\%\text{Inhibition}=\left[\left(A_{\text{control}}-A_{\text{Sample}}\right)/A_{\text{control}}\right]\times 100$$

A, Absorbance value at 734 nm.

#### 2.8.2 DPPH ^•^ scavenging activity

The DPPH radical scavenging activities of samples were assayed according to the Blois method. Blois (1958) 1 mM DPPH^•^ solution was used as free radical, whereas *α*‐tocopherol and trolox were used as standard antioxidants. Stock solutions were prepared at 10 different concentrations (5–100 μg/mL) of the extracts and standards. The absorbances of whole samples were read at 517 nm against the blank consisting of absolute ethanol. All measurements were repeated three times to ensure accuracy and precision in the study.

The DPPH^•^ radical scavenging capacity was calculated according to the following equation:

$$\%\text{Inhibition}=\left[\left(A_{\text{control}}-A_{\text{Sample}}\right)/A_{\text{control}}\right]\times 100$$

A, Absorbance value at 517 nm.

#### 2.8.3 Total phenolic content

**FIGURE 1:**
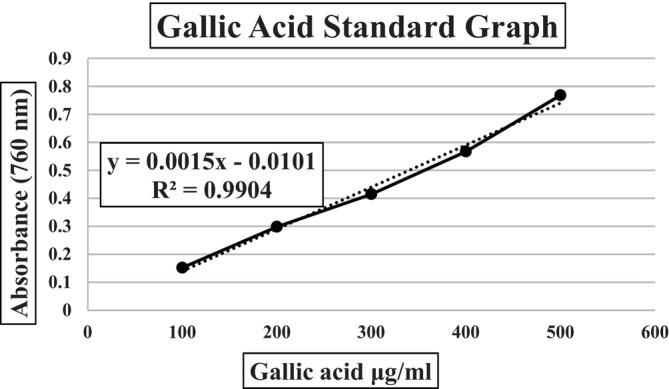
The gallic acid standard graph. [(760 nm) Absorbance = 0.0015 × Gallic acid – 0.0101].

Total phenolic content of the samples was detected using the method developed by Folin & Denis (1912) and modified by Slinkard & Singleton (1977). Gallic acid (GA) was chosen as the standard. Gallic acid solutions prepared in the concentration range of 100 and 700 μg/mL were treated with Folin–Ciocalteu reagent (FCR) and aqueous Na_2_CO_3_. The gallic acid standard graph was obtained by recording absorbances at 760 nm (blank: distilled water). Samples were treated the same as standard solutions and absorbances were recorded at 760 nm. The absorbances of the samples were substituted in the equation of Absorbance = 0.0015 × Gallic acid – 0.0101 obtained from the standard graph (760 nm), and the total amount of phenolic components was calculated in gallic acid equivalents (GAE) (Figure 1). Each analysis was repeated three times.

#### 2.8.4 Total tannin content

**FIGURE 2:**
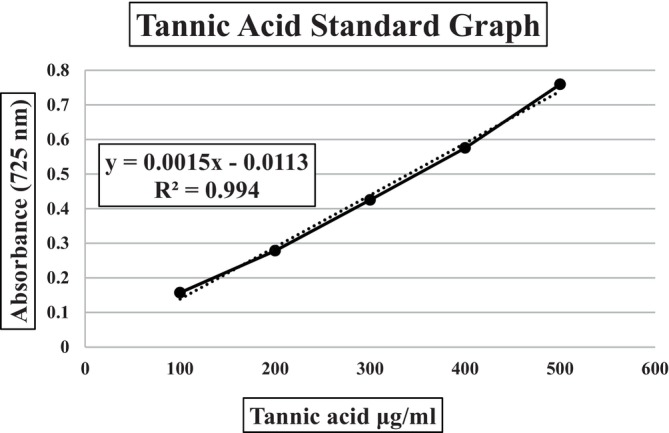
The tannic acid standard graph. [(725 nm) Absorbance = 0.0015 × Tannic acid – 0.0113].

Total tannin content of the samples was determined based on the method modified by Makkar according to the Folin–Ciocalteu method (Makkar & Makkar, 2003). Tannic acid was chosen as standard. Tannic acid solutions prepared in the concentration range of 100 and 700 μg/mL were treated with Folin–Ciocalteu reagent (FCR) and aqueous Na_2_CO_3_. Absorbances of the samples were read at 725 nm against a blank consisting of distilled water. Measurements were repeated three times. The tannic acid equivalents (TAE) corresponding to the absorbance values of the samples were found with the help of the equation obtained from the standard graph (Figure 2). Results are given in tannic acid equivalents (TAE) and micrograms.

### 2.9 Antimicrobial activity (MIC, μg/mL)

Antimicrobial activity of the samples was evaluated against *Escherichia coli* ATCC 8739, *Staphylococcus aureus* ATCC 6538, *Pseudomonas aeruginosa* ATCC 9027, *Candida tropicalis* ATCC 750, and *Candida albicans* ATCC 90028. The beginning concentrations of the extracts were prepared at (9.79–5000 μg/mL). The extracts were prepared within dimethyl sulfoxide (10% of DMSO) and water. Standards used were cefuroxime and fluconazole at 0.25–128 μg/mL within DMSO and water.

Antibacterial and antifungal susceptibility tests were evaluated using modified protocols on microdilution technique for aerobic microorganisms (M100‐S16) and fungi (M27‐A2) published by Clinical and Laboratory Standards Institute (CLSI) or former National Committee for Clinical Laboratory Standards (NCCLS) (Clinical and Laboratory Standards Institute [CLSI], 2006, 2008).

To refresh cultures, they were streaked on petri dishes containing Mueller–Hinton Agar (MHA), Potato Dextrose Agar (PDA), or Sabouraud Dextrose Agar (SDA), and incubated at 37°C for 24 h. After incubation, single colonies grown on agar plates were transferred to tubes containing Mueller–Hinton Broth (MHB) (Roswell Park Memorial Institute (RPMI) medium for *Candida* species) and incubated at 37°C for 24 h. Following the 18–24 h incubation period, the cultures in liquid medium were adjusted to a turbidity corresponding to McFarland No. 0.5 (approximately 10^8^ CFU/mL (colny‐forming units per milliliter) for bacteria and 10^6^ CFU/mL for yeast cultures) using a turbidimeter. Positive growth controls were performed in wells not containing antimicrobial agents. MIC was determined visually as the lowest concentration of the extract at which no bacterial growth was visible. Antimicrobial activity tests were performed as three replicates and the mean of tests represented as MIC.

## 3 RESULTS AND DISCUSSION

The personal characteristics of pharmacists are outlined in Table 1. Among the pharmacist population, 57.1% were female, 71.4% held university degrees, and 31.4% had 0–5 years of experience in the profession, and 57.1% had their pharmacies around the Health Center–Family Health Center (FHC)–Community Health Center (CHC)–Hospital vicinity. Notably, the proportion of individuals aged 26–30 years is significantly higher (28.7%) compared to other age groups, while the participation of those aged 21–25 years is notably low (Table 1).

**TABLE 1:** Demographic characteristics of community pharmacists (*n* = 35).

Variables	*N*	(%)
Gender		
Female	15	42.9
Male	20	57.1
Age		
21–25	2	5.7
26–30	10	28.7
31–35	9	25.8
36–40	5	13.9
41 and over	9	25.9
Educational level		
University	25	71.4
Master's degree	6	17.2
Doctorate	4	11.4
Years of seniority in the profession		
0–5	11	31.4
6–10	6	17.2
11–15	9	25.7
16–20	2	5.7
≥21	7	20.0
Location of the pharmacy		
Health center–FHC–CHC–Hospital vicinity pharmacy	20	57.1
Neighborhood Pharmacy	15	42.9

Abbreviations: CHC, Community Health Center; FHC, Family Health Center.

As shown in Table 2, the sale of herbal supplements (68.6%) is prevalent in pharmacies, and the specific products include Ginkgo biloba, Hametan cream, Linden–Chamomile tea, Black Elderberry, Passiflora, Garlic (20.2%), Peanut oil, and Black cumin/oil/capsule. The percentage of pharmacists selling herbal products containing garlic in the pharmacy is 51.4% (*n* = 18). Among the garlic products sold in pharmacies, the most common are fermented garlic supplements (tablets, capsules, etc.) (black garlic) (33.3%) and shampoo (33.3%). The garlic products sold in pharmacies are predominantly reported to be based on patient requests (66.7%). It has been reported that products sold in pharmacies are predominantly purchased by young patients aged 19–64 years (72.2%) and male patients (77.7%). Furthermore, these products are primarily sold to patients with high blood pressure and baldness. Pharmacists mainly reported that they receive questions from patients regarding garlic products (77.7%) and that they are not particularly concerned (72.2%) about these products (Table 2).

**TABLE 2:** Answers on form on the sales practice of garlic products.

Question	Answer	*N* (%)
Which types of herbal products do you sell in your pharmacy?^a^	Herbal supplements	24 (68.6)
	Herbal teas	5 (14.2)
	Vegetable oils	7 (20)
	Fresh herbs and plants	1 (2.9)
Which herbal product (containing a single plant) is most in demand in your pharmacy?	Hawthorn vinegar olive oil	1 (5.5)
	Ginkgo biloba	4 (11.4)
	Hametan cream	4 (11.4)
	Linden–Chamomile tea	4 (11.4)
	Black Elderberry	4 (11.4)
	Passiflora	4 (11.4)
	Garlic	7 (20.2)
	Peanut oil	4 (11.4)
	Black cumin/oil/capsule	4 (11.4)
Do you sell garlic products in your pharmacy?	Yes	18 (51.4)
	No	17 (48.6)
Which types of garlic products do you sell in your pharmacy?^a^	Fermented garlic supplements (tablets, capsules, etc.) (black garlic)	6 (33.3)
	Shampoo	6 (33.3)
	Garlic supplements (tablets, capsules, etc.)	2 (11.0)
	Garlic oil	4 (22.4)
Give examples of your best‐selling garlic preparations^a^	Bioxcin black garlic shampoo	16 (80.0)
	Solgar garlic oil	4 (20.0)
In terms of supplying garlic products to patients; from whom is the demand coming?	From patient	12 (66.7)
	From pharmacist	6 (33.3)
What is the patient profile to whom you sell garlic products the most?	Young patients	13 (72.2)
	Old patients	5 (27.8)
What is the patient group you sell garlic products the most?	Blood pressure patients	7 (38.9)
	Patients using for Alopesia areata	6 (33.3)
	Patients using for prophylactic (preventive) purposes	5 (27.8)
What is the gender of the patient group you sell garlic products the most?	Female	4 (22.3)
	Male	14 (77.7)
Do you receive questions from patients about garlic products?	Yes	14 (77.7)
	No	4 (22.3)
Do you have any concerns or worries about garlic products being sold in pharmacies?	Yes	5 (27.3)
	No	13 (72.2)

^a^ More than one option is marked.

Sample 1 essential oil, constituting 91.7% of the sample, comprised 32 identified compounds. The major constituents included allyl methyl trisulfide (17.5%), diallyl disulfide (11.5%), dimethyl trisulfide (10.3%), acetic acid (8.0%), and allyl methyl disulfide (7.8%). Sample 2 essential oil, constituting 91.8% of the sample, contained 10 identified compounds. The primary constituents were diallyl disulfide (26.8%), diallyl sulfide (19.1%), allyl methyl disulfide (9.5%), and allyl methyl trisulfide (10.0%). Sample 3 essential oil, representing 85.4% of the sample, comprised 16 compounds. The main constituents were diallyl disulfide (48.6%), allyl methyl disulfide (9.3%), and 3‐vinyl‐1,2‐dithi‐4‐ene (6.1%). Sample 4 essential oil, constituting 75.0% of the sample, contained 30 identified compounds. The major constituents included tridecane (8.0%), diallyl disulfide (15.4%), and diallyl sulfide (5.5%). Sample 5 essential oil, representing 92.8% of the sample, comprised 12 identified compounds. The main constituents were allyl methyl disulfide (9.8%), diallyl disulfide (33.5%), allyl methyl trisulfide (13.6%), and 3‐vinyl‐1,2‐dithi‐4‐ene (10.2%). Sample 7 essential oil, containing 88.8% of the sample, comprised 37 identified compounds. The main constituents were acetic acid (28.6%), isovaleric acid (8.2%), and allyl methyl trisulfide (8.0%). Sulfur compounds were the major group in Samples 1, 2, 3, 5, 7, and 8, with a range of 23.3%–91.6%. Volatile components of garlic samples analyzed using the SPME method are given in Table 3.

**TABLE 3:** Volatile components of garlic samples analyzed using the SPME method.

No	RRI	Component	1%	2%	3%	4%	5%	7%	8%
1	1083	Dimethyl sulfide	–	–	–	–	–	0.2	–
2	1159	Diallyl sulfide	–	19.1	3.1	–	5.5	4.6	0.4
3	1200	Dodecane	–	–	–	–	0.9	–	–
4	1203	Limonene	–	–	–	–	2.1	–	–
5	1274	Methyl *cis*‐propenyl disulfide	–	–	–	–	–	–	0.2
6	1280	*p*‐Cymene	–	–	–	–	0.9	–	–
7	1292	Allyl methyl disulfide	7.8	9.5	9.3	–	2.9	9.8	5.5
8	1294	Methyl *trans*‐propenyl disulfide	–	–	–	–	–	0.4	0.1
9	1300	Tridecane	–	–	–	–	8.0	–	–
10	1332	2,5‐Dimethyl pyrazine	–	–	–	–	–	0.3	–
11	1350	Anisol	–	–	–	–	–	–	0.3
12	1360	1‐Hexanol	–	–	–	–	0.7	–	–
13	1362	Ethyl lactate^a^	–	–	–	2.6	–	–	–
14	1393	Dimethyl trisulfide	10.3	5.8	–	0.1	–	2.8	3.6
15	1400	Tetradecane	–	–	–	0.2	1.8	–	–
16	1438	Allyl propenyl disulfide	–	0.4	1.6	–	–	0.7	–
17	1475	Acetic acid*	8.0	–	–	3.6	–	–	28.6
18	1479	Furfural	2.4	–	–	10.1	–	–	6.2
19	1492	Diallyl disulfide*	11.5	26.8	48.6	–	15.4	33.5	4.9
20	1496	2‐Ethyl hexanol	–	–	–	4.4	–	–	–
21	1516	2‐Acetyl furan	0.8	–	–	2.4	–	–	3.2
22	1522	Unknown (M^+^146)	0.5	–	6.3	–	–	2.9	–
23	1541	Benzaldehyde	0.1	–	–	0.3	–	–	1.5
24	1562	Propanoic acid	–	–	–	0.2	–	–	3.7
25	1591	2‐Methyl propanoic acid	–	–	–	–	–	–	1.0
26	1565	Unknown 1 (Bp 103)	12.7	13.9	3.6	–	11.7	11.9	–
27	1585	5‐Methyl furfural	0.9	–	–	3.2	–	–	2.2
28	1600	Hexadecane	–	–	–	–	1.2	–	–
29	1607	Allyl methyl trisulfide*	17.5	10.0	2.4	0.4	3.0	13.6	8.0
30	1631	Butanoic acid	–	–	–	–	–	–	0.4
31	1638	Ethyl levulinate^a^, *	–	–	–	12.9	–	–	–
32	1651	*γ*–Butyrolactone	tr	–	–	–	–	–	0.6
33	1668	Furfuryl alcohol	0.5	–	–	3.4	–	–	0.3
34	1684	Isovaleric acid	0.5	–	–	0.7	–	–	8.2
35	1706	α‐Terpineol	–	–	–	–	0.4	–	–
36	1726	*γ*‐Hexalactone	0.3	–	–	–	–	–	tr
37	1762	Pentanoic acid	–	–	–	0.5	–	–	–
38	1772	Methoxy phenyl oxime^a^	–	–	–	–	–	–	2.2
39	1783	3‐Vinyl‐1,2‐dithi‐4‐ene	4.4	3.8	6.1	–	1.8	10.2	–
40	1786	*ar*‐Curcumene	–	–	–	–	0.3	–	–
41	1811	Diallyl trisulfide	1.3	2.3	1.5	tr	0.4	1.8	0.6
42	1856	*Dipropylene glycol* ^b^	–	–	–	–	0.5	–	–
43	1857	Geraniol	–	–	–	3.0	–	–	–
44	1870	Hexanoic acid	0.3	0.2	–	5.6	1.4	0.1	2.9
45	1878	Guaiacol	–	–	–	–	0.3	–	–
46	1896	Benzyl alcohol	0.4	–	–	–	–	–	–
47	1937	Phenyl ethyl alcohol	–	–	–	1.2	–	–	–
48	1981	Heptanoic acid	0.3	–	–	1.1	–	–	0.2
49	2012	2‐Acetylpyrrole	0.5	tr	–	0.7	0.2	–	0.6
50	2065	*γ*‐Nonalactone	–	–	–	3.1	–	–	–
51	2084	Octanoic acid	0.4	–	–	3.1	0.4	–	–
52	2096	(*E*)‐Methyl cinnamate	0.1	–	tr	0.2	tr	–	0.4
53	2186	Eugenol	–	–	–	–	0.3	–	0.1
54	2192	Nonanoic acid	0.6	–	tr	2.9	0.4	–	0.3
55	2239	Carvacrol	–	–	0.4	1.8	–	–	–
56	2260	Ethyl hexadecanoate	–	–	–	1.8	–	–	–
57	2298	Decanoic acid	0.3	–	–	–	–	–	0.1
58	2300	Tricosane	0.2	–	0.1	–	–	–	–
59	2308	Methyl dihydrojasmonate	–	–	–	0.7	–	–	–
60	2400	Tetracosane	0.7	–	0.2	–	–	–	0.2
61	2500	Pentacosane	1.3	–	0.5	–	–	–	0.6
62	2530	Benzoic acid	0.3	–	–	4.1	–	–	0.5
63	2600	Hexacosane	1.8	–	0.6	–	–	–	0.6
64	2670	Tetradecanoic acid	–	–	–	0.7	–	–	–
65	2700	Heptacosane	1.7	–	0.6	–	–	–	0.6
66	2800	Octacosane	1.4	–	0.5	–	–	–	tr
67	2900	Nonacosane	1.2	–	–	–	–	–	tr
68	2931	Hexadecanoic acid	0.7	–	–	–	–	–	tr
		Monoterpene hydrocarbons	–	–	–	–	3.0	–	–
		Oxygenated monoterpenes	0.1	–	0.4	5.0	0.7	–	0.5
		Sesquiterpene hydrocarbons	–	–	–	–	0.3	–	–
		Oxygenated sesquiterpenes	–	–	–	–	0.3	–	–
		Sulfur compounds	66	91.6	82.5	0.5	40.7	92.4	23.3
		Fatty acid + esters	3.1	0.2	–	16.6	2.2	0.1	16.8
		Alkanes + Alkenes	8.3	–	2.5	0.2	11.9	–	2.0
		Others	14.2	–	–	52.7	1.4	0.3	46.2
		Total	91.7	91.8	85.4	75.0	60.5	92.8	88.8

*Note*: Unknown I: EIMS, 70 eV, m/z (rel. .int.): 103[M]^+^(100), 78(2), 71(8), 69(8), 64(6), 59(6), 45(16), 39(6). Unknown II: EIMS, 70 eV, m/z (rel. .int.): 138[M]^+^(100), 96(12), 74(46), 73(44), 64(15), 59(14), 45(16), 41(26), 39(10). % calculated from total ion chromatogram (TIC).

Abbreviations: RRI, Relative retention indices calculated against *n*‐alkanes; tr, Trace (<0.1%).

^a^ Tentative identification from Wiley.

^b^ Solvent (as a carrier).

* Major compounds.

Sample 6 essential oil, comprising 98.9% of the sample, was identified to contain 23 compounds. The primary compounds included linoleic acid (38.0%) and oleic acid (36.8%), together constituting a significant portion of the composition. In Sample 7, 22 compounds, totaling 98.9%, were identified. Linoleic acid emerged as the predominant compound at 62.8%, followed by oleic acid at 16.8%. These two compounds constituted the majority of the essential oil composition in Sample 7. Fatty acids and esters were the predominant group in Samples 6 and 7, with concentrations of 92.8% and 96.6%, respectively. Chemical composition of the methylated garlic in Samples 6 and 7 is provided in Table 4.

**TABLE 4:** Chemical composition of the methylated garlic in Samples 6 and 7.

No	RRI	Component	6%	7%
1	1213	1,8‐Cineole	2.1	1.0
2	1255	*γ*‐Terpinene	tr	tr
3	1280	*p*‐Cymene	tr	tr
4	1532	Camphor	0.5	0.2
5	1590	Bornyl acetate	tr	tr
6	1612	*β*‐Caryophyllene	1.4	tr
7	1614	Carvacrol methyl ether	tr	tr
8	1694	Neral	tr	0.1
9	1719	Borneol	0.3	tr
10	1726	Germacrene D	0.3	0.1
11	1740	Geranial	tr	tr
12	1755	Bicyclogermacrene	tr	tr
13	1773	δ‐Cadinene	tr	–
14	1808	Nerol	tr	tr
15	1857	Geraniol	0.4	0.3
16	2198	Thymol	0.4	0.2
17	2239	Carvacrol	0.7	0.4
18	2931	Hexadecanoic acid	11.0	11.3
19	3150	Octadecanoic acid	4.0	4.5
20	3200	Oleic acid*	36.8	16.8
21	3210	Elaidic acid	2.0	0.7
22	3290	Linoleic acid*	38.0	62.8
23	3300	Linolenic acid	1.0	0.5
		Monoterpene hydrocarbons	tr	tr
		Oxygenated monoterpenes	4.4	2.2
		Sesquiterpene hydrocarbons	1.7	0.1
		Fatty acid + esters	92.8	96.6
		Total	98.9	98.9

*Note*: % calculated from total ion chromatogram (FID).

Abbreviations: RRI, Relative retention indices calculated against *n*‐alkanes; tr, Trace (<0.1%).

* Major compunds.

Diallyl disulfide (DADS) was found at its highest concentration in Samples 3 and 7, specifically in the directly cultivated and garlic oil pearls. However, it was notably absent in Sample 4. Also, allyl methyl disulfide was found at its highest concentration in Samples 2 and 7, specifically in the dried and garlic oil pearls. However, it was notably absent in Sample 4.

Air‐dried, oven‐dried, and freeze‐dried garlic bulbs underwent hydrodistillation, and the resultant essential oils were subjected to analysis through GC and GC/MS. The essential oils exhibited a notable presence of sulfur compounds, ranging from 84.3% to 98.9%. Diallyl trisulfide emerged as a predominant component, constituting 37.3%–45.9%, followed by diallyl disulfide at 17.5%–35.6% and methyl allyl trisulfide at 7.7%–10.4%. A distinctive set of marker components was identified to facilitate differentiation between the essential oils derived from air‐dried, oven‐dried, and freeze‐dried garlic bulbs (Dziri et al., 2014). The chemical makeup of garlic essential oils obtained from two distinct cultivars was analyzed through GC–MS analysis. Essential oil extracted from the white‐skin cultivar exhibited lower proportions of key constituents, namely diallyl trisulfide and diallyl disulfide, accounting for 45.76% and 15.63%, respectively. In contrast, the purple‐skin cultivar displayed higher percentages of the same components, amounting to 58.53% and 22.38%, respectively (El‐Sayed et al., 2017). In the effort to categorize authentic garlic cultivars, an investigation into the oil composition and genetic characteristics of three commonly utilized garlic cultivars for essential oil production in the northern Thai market was conducted. Essential oils were extracted through hydrodistillation and microwave hydrodistillation, and their chemical components were subsequently analyzed using gas chromatography–mass spectrometry. The principal compounds identified in these essential oils included the trisulfide, di‐2‐propenyl, the disulfide, di‐2‐propenyl, and the trisulfide, methyl 2‐propenyl (Somamno et al., 2016).

Antioxidant activity of the samples (fermented (1), dried sample (2), directly cultivated sample (3), garlic extract tablets (4), capsules (5), garlic oil (6), garlic oil pearls (7), and fermented garlic (8) results) is presented in Table 5. In ABTS cation radical scavenging tests, when the percentage (%) inhibition values of the standards (*α*‐tocopherol (TK), trolox (TR)) and samples were compared at a concentration of 50 μg/mL, it was determined that standards had a higher % inhibition value on ABTS^·+^. If the samples and standards were evaluated in terms of ABTS^·+^ scavenging capacity, we can conclude that Sample 1 showed higher % inhibition than other samples, although not as much as the standards [(TR)52.2 > (TK)23.3 > (1)9.3 > (3)7.2 > (2)6.6 > (5)2.8 > (7)2.2 > (4)1.2 > (6)0.9 > (8)0.3%; 50 μg/mL % inhibition].

**TABLE 5:** Antioxidant activity test results.

	ABTS^•+^ scavenging activity (% inhibition of 50 μg/mL ± standard deviation)	DPPH^•^ scavenging activity (% inhibition of 50 μg/mL ± standard deviation)
Standards		
*α*‐Tocopherol	23.310 ± 0.0002	64.955 ± 0.0164
Trolox	52.290 ± 0.0112	92.940 ± 0.0229
Samples		
3	7.273 ± 0.0146	3.043 ± 0.0006
2	6.625 ± 0.0098	2.541 ± 0.0063
1	9.329 ± 0.0075	3.665 ± 0.0202
4	1.219 ± 0.0056	1.251 ± 0.0062
5	2.819 ± 0.0047	1.558 ± 0.0041
6	0.923 ± 0.0054	1.149 ± 0.0239
7	2.222 ± 0.0143	1.356 ± 0.0044
8	0.341 ± 0.0098	1.056 ± 0.0620

In DPPH radical scavenging tests, when the % inhibition values of the standards and samples were compared at a concentration of 50 μg/mL, it was determined that standards had a higher % inhibition value on DPPH^•^. If the samples and standards were evaluated in terms of DPPH^•^ scavenging capacity, we can conclude that that Sample 1 showed higher % inhibition value than other samples, although not as much as the standards [(TR)92.9 > (TK)64.9 > (1)3.6 > (3)3.0 > (2)2.5 > (5)1.5 > (7)1.3 > (4)1.2 > (6)1.1 > (8)1.0%; 50 μg/mL % inhibition]. Other studies have shown that the antioxidant effects and nutritional values of fermented garlic are increased compared to raw garlic (Tahir et al., 2022). In this regard, the results of antioxidant activity tests support each other and literature.

Garlic, which is used as a spice as well as to treat and prevent various ailments, was discovered thousands of years ago. It has been shown to be particularly beneficial in cardiovascular and microorganism‐related disorders, as well as anticancer effects (Londhe et al., 2011). Today, various forms of garlic have been created and used. This study, which compares several features as well as antioxidant activity of various types of garlic, is useful in the literature.

Total phenolic and tannin content of extracts test results is presented in Table 6. When the studies on the extracts were evaluated, it was concluded that especially Sample 3 was richer in total phenol and total tannin content. The results of total phenolic and tannin content experiments were similar to the antioxidant activity tests results.

**TABLE 6:** Total phenolic and tannin content of samples.

	Total phenolic compound (μg GAE/mg extract ± standard deviation)	Total tannin compound (μg TAE/mg extract ± standard deviation)
Samples		
3	11.555 ± 0.0007	12.355 ± 0.0007
2	10.2 ± 0.0009	11.0 ± 0.0009
1	13.866 ± 0.0002	14.666 ± 0.0002
4	9.066 ± 0.0018	9.866 ± 0.0018
5	9.955 ± 0.0014	10.755 ± 0.0014
6	8.435 ± 0.0002	9.235 ± 0.0002
7	9.154 ± 0.0003	9.954 ± 0.0003
8	7.679 ± 0.0016	8.479 ± 0.0016

Diallyl disulfide (DADS), an organosulfur compound derived from garlic, was examined for its protective properties, antioxidant capabilities, and anti‐inflammatory effects against cyclophosphamide (CP)‐induced hepatotoxicity in rats. The results suggest that the administration of DADS has the potential to alleviate CP‐induced liver damage by simultaneously enhancing antioxidant defenses and mitigating inflammation (Hasan et al., 2020). Also, DADS is known for its antioxidant properties and demonstrates a protective effect on the kidneys in the experimental model (Pedraza‐Chaverrí et al., 2003). As seen in our results, Samples 1 and 3, which carry DADS at the highest rate, showed the highest antioxidant effects.

According to the data presented in the tables, the samples were evaluated for their antidiabetic and anticholinesterase activities, with inhibition percentages provided at specific concentrations. Notably, Sample 6 (garlic oil) exhibited the highest *α*‐glucosidase inhibition among the antidiabetic activities, reaching 28.93%, while Sample 1, Sample 4, and Sample 5 exhibited no discernible effects. Acarbose, the positive control, displayed a substantial 40.86% inhibition (Table 7).

**TABLE 7:** In vitro antidiabetic and anticholinesterase activities of garlic products, natural and fermented garlics.

Samples	Antidiabetic activity		Anticholinesterase activity	
	*α*‐Glucosidase inhibition (%) (1000 μg/mL) (mean ± std)	*α*‐Amylase inhibition (%) (5000 μg/mL) (mean ± std)	Acetylcholinesterase inhibition (%) (100 μg/mL) (mean ± std)	Butyrylcholinesterase inhibition (%) (1000 μg/mL) (mean ± std)
1	N.D.	8.31 ± 0.71	12.85 ± 3.33	6.62 ± 3.98
2	2.53 ± 3.93	41.27 ± 0.33	14.60 ± 3.47	9.02 ± 4.13
3	3.26 ± 0.17	46.84 ± 1.59	19.74 ± 4.63	7.61 ± 6.95
4	N.D.	12.03 ± 5.50	15.38 ± 2.78	5.06 ± 4.24
5	N.D.	51.50 ± 6.57	22.39 ± 5.82	11.66 ± 7.64
6	28.93 ± 4.56	48.05 ± 0.38	12.75 ± 1.75	3.88 ± 5.16
7	12.77 ± 6.41	45.77 ± 2.22	22.92 ± 0.94	13.37 ± 3.81
8	1.92 ± 1.86	12.46 ± 1.23	19.66 ± 0.37	12.47 ± 1.41
Acarbose^a^	40.86 ± 4.04	68.89 ± 1.30	–	–
Donepezil^b^	–	–	99.81 ± 0.64	100 ± 0.53

Abbreviation: N.D., Not determined.

^a^ Positive control for antidiabetic activity.

^b^ Positive control for anticholinesterase activity.

In terms of *α*‐amylase inhibition, Sample 5 (capsules) demonstrated the highest efficacy at 51.50%, followed by Sample 6 (garlic oil) with 48.05% inhibition. Conversely, Sample 1 (fermented) had the lowest *α*‐amylase inhibitory effect at 8.31%.

A research focused on the inhibitory effect of garlic extract (diallyl trisulfide) (in 100 mg of garlic oil) on *α*‐glucosidase. Garlic extracts demonstrated a dose‐dependent inhibition comparable to the pharmaceutical acarbose, with an IC_50_ value of 16.93 mg/mL, while acarbose's IC_50_ value was 3.19 mg/mL as similar as our study (Obih et al., 2019).

Another study investigated the impact of convective hot‐air drying on inhibitory properties of garlic against *α*‐amylase and *α*‐glucosidase enzymes. Thin garlic samples were single‐layer chopped and subjected to drying at temperatures ranging from 50 to 80°C, with specific humidity levels of 10 and 20 g water per kilogram (kg) of dry air, and a constant air velocity of 0.5 m/s using an ICT‐controlled lab dryer. The results indicated that fresh garlic exhibited the highest *α*‐amylase inhibition (15.81%), but as drying temperatures increased, the *α*‐amylase inhibition capacity decreased. Interestingly, the specific humidity levels of dry air did not significantly affect the *α*‐amylase inhibition activities in dried garlic samples. The impact of hot‐air drying temperature on *α*‐glucosidase inhibition activities mirrored that on *α*‐amylase. Notably, garlic samples dried at a specific humidity of 20 g water/kg dry air displayed a high percentage of *α*‐glucosidase inhibition, with the range for both *α*‐amylase and *α*‐glucosidase inhibition varying from 7.81% to 15.81% and 5.42% to 13.63%, respectively. (Wongsa et al., 2023) The study examined the impacts of garlic oil and diallyl disulfide DADS on glycemic control and renal function in rats induced with streptozotocin‐induced diabetes. Rats were orally administered garlic oil or DADS every other day for 16 weeks after diabetes induction (Liu et al., 2006). Antidiabetic effect of garlic oil but not diallyl disulfide was observed in rats with streptozotocin‐induced diabetes. The objective was to discover and assess natural organosulfur compounds (OSCs) from the *Allium* genus as effective dual inhibitors of both α‐amylase and α‐glucosidase through a series of molecular simulations and in vitro analyses. In silico screening demonstrated robust interactions between OSCs and the crystal structures of α‐amylase and α‐glucosidase (Ahmad et al., 2021).

In summary, the prolonged administration of garlic oil in diabetes treatment exhibited enhancements in oral glucose tolerance and renal function, contrasting with the ineffectiveness of DADS in achieving similar outcomes. Elevated doses of DADS were found to potentially exacerbate metabolic disturbances in diabetes.

Our study also yielded similar results for fresh (3.26% *α*‐glucosidase inhibition and 46.84% *α*‐amylase inhibition) and hot‐dried (2.53% *α*‐glucosidase inhibition and 41.27% *α*‐amylase inhibition) samples.

Moving on to anticholinesterase activities, Sample 7 (garlic oil pearls) exhibited the most notable inhibition against both acetylcholinesterase (22.92%) and butyrylcholinesterase (13.37%). On the other hand, Sample 6 (garlic oil) displayed the least effectiveness with 12.75% acetylcholinesterase inhibition and 3.88% butyrylcholinesterase inhibition. Donepezil exhibited potent anticholinesterase effects, with nearly complete inhibition for both acetylcholinesterase (99.81%) and butyrylcholinesterase (100%).

A study investigated the impact of garlic essential oil (GEO) on acetylcholinesterase (AChE) activity. The study explored the interactions of garlic essential oil with AChE activity in cerebral cortex synaptosomes. Results indicated that garlic essential oil inhibited AChE activity in a dose‐dependent manner (0–50 μg/mL). These effects were attributed to the presence of monoterpenes in garlic essential oils. However, the observed inhibitory effect of garlic oil (IC_50_ = 43.5 μg/mL) on AChE was less potent than the well‐known cholinesterase inhibitor, donepezil (IC_50_ = 0.65 μg/mL) (Akinyemi et al., 2018). Another study aimed to explore the neuroprotective effects of essential oil from *Allium sativum* cloves against the elevation of cholinesterase enzymes induced by β‐Amyloid 25–35 (Aβ25‐35) in Alzheimer's disease (AD). The essential oil demonstrated lower anticholinesterase activity, with inhibition percentages of 65.4% for acetylcholinesterase (AChE) and 31.5% for butyrylcholinesterase (BChE) (Ghajarbeygi et al., 2019). The methanol extracts of *Allium tuncelianum* were subjected to analysis using liquid chromatography–mass‐spectrometry (LC–MS/MS) to determine their phytochemical composition. Anticholinesterase and antidiabetic activities were evaluated through enzyme inhibition assays, specifically targeting AChE and α‐glucosidase enzymes. The results revealed significant inhibitory effects on both AChE (IC_50_: 11.25 μg/mL) and α‐glucosidase (IC_50_: 9.85 μg/mL) enzymes. This suggests that *A. tuncelianum* could serve as a promising alternative treatment for diabetes and certain neurodegenerative diseases (Takim et al., 2021). Allicin, an organosulfur compound derived from the metabolism of alliin in garlic, has been documented to function as an indirect muscarinic agonist. This is achieved through its inhibitory effects on acetylcholinesterase and butyrylcholinesterase. By inhibiting these cholinesterase enzymes, allicin reduces the breakdown rate of acetylcholine into choline, ultimately leading to an increase in parasympathetic nervous stimulation (Alare et al., 2021). AD stands as the prevailing neurodegenerative disorder. Garlic is believed to exert diverse physiological effects, potentially playing a protective role against dementia. The study delved into examining the inhibitory potential of garlic essential oil (GEO) against enzymes associated with AD and assessing the distribution of active compounds in GEO within the brain. The results indicated that numerous sulfur compounds in GEO demonstrated significant inhibition of enzymes linked to AD. Furthermore, these sulfur compounds were detected in both serum and the brain 6 h after administration (Yoshioka et al., 2021).

This study represents the inaugural comprehensive evaluation of various formulations of garlic products.

In vitro antimicrobial activities of the garlic products, natural and fermented garlics are presented in Table 8. Generally, the MIC value was observed to be between 625 and 5000 μg/mL for the samples. In our study, Samples 3, 6, and 8 were found to be more effective against *C. tropicalis* with MIC = 625 μg/mL. Sample 7 was found to be more effective against *Candida* yeast than the bacteria with MIC = 1250 μg/mL.

**TABLE 8:** In vitro antimicrobial activities of garlic products, natural and fermented garlics (MIC, μg/mL).

Extracts	*E. coli* ATCC 8739	*S. aureus* ATCC 6538	*P. aeruginosa* ATCC 9027	*C. tropicalis* ATCC 750	*C. albicans* ATCC 90028
1	2500	2500	2500	5000	5000
2	>1250	>1250	>1250	2500	2500
3	>1250	>1250	>1250	625	2500
4	>2500	2500	>2500	2500	5000
5	>1250	>1250	>1250	2500	>2500
6	>1250	>1250	>1250	625	>2500
7	>1250	>1250	>1250	1250	1250
8	2500	2500	2500	625	1250
Cefuroxime	32	32	64	–	–
Fluconazole	–	–		8	16

*Note*: Fermented (1), dried sample (2), directly cultivated sample (3), garlic extract tablets (4), capsules (5), garlic oil (6), garlic oil pearls (7), and fermented garlic (8).

Dialyl thiosulfinate or diallyl disulfide, also known as allicin, is one of the most biologically active substances found in garlic (Gebreyohannes & Gebreyohannes, 2013). Major compounds such as diallyl sulfide, allyl methyl disulfide, dimethyl trisulfide, diallyl disulfide, and allyl methyl trisulfide, 3‐vinyl‐1,2‐dithi‐4‐ene, and diallyl trisulfide are among the volatile components of Samples 3–7–8 that were examined using the SPME method. The primary compounds included linoleic acid (38.0%) and oleic acid (36.8%) for Sample 6 oil.

A study investigated that the most effective garlic powder tablets exhibited an activity level comparable to that of clove homogenates, while steam‐distilled oils demonstrated an activity level of 35%, and oil‐macerates, owing to their lower content, only 12%. Diallyl trisulfide was primarily responsible for the activity in steam‐distilled oils. The vinyl dithiins were primarily responsible for the majority of the activity in the oil‐macerates. Ajoene, which is only found in oil‐macerates, exhibited the highest specific activity among all the compounds tested. However, due to its low concentration, it only possessed 13% of diallyl trisulfide's activity and 3% of allicin's activity (Lawson et al., 1992).

A study investigated that several commercial preparations of garlic, which included oil‐macerates, pastes, tablets, and powders, were examined for their anticandidal activity against *C. albicans*. Type 4 (a tablet of garlic freeze‐dried) was found to be the most active commercial preparation of garlic over the 6 h test period. Type 4 had approximately 20 times higher total thiosulfinate concentration. All the other types (Pearls, capsule–oil, Softgel, capsule–oil‐macerate, Garlic gems, capsule–powder, Enteric coated, capsule–liquid, Pearls, capsule–paste, Pearls, capsule–oil, Pearls, capsule–powder, Pearls, capsule–powder, and Pearls, capsule–oil extract) did not inhibit *C. albicans* (Elsom et al., 2003).

In a 56‐patient randomized trial, topical application of garlic paste for 14 days suppressed clinical signs of oral candidiasis in addition to clotrimazole solution. The results of this preliminary study discuss the possible application of garlic paste in the treatment of oral candidiasis (Sabitha et al., 2005).

## 4 CONCLUSIONS

In summary, this comprehensive study on garlic products, including popular items from Turkish pharmacies and those grown under standard conditions, revealed diverse chemical compositions and multifaceted health properties. Notably, black garlic extract tablets, garlic oil, and fermented garlic emerged as prevalent products. The chemical analysis highlighted key compounds like diallyl disulfide, allyl methyl disulfide, and allyl methyl trisulfide in varying concentrations. Sample 1 displayed superior antioxidant activity, while Sample 3 exhibited richness in total phenol and total tannin content. Additionally, Sample 6 demonstrated notable α‐glucosidase inhibition, while Sample 5 capsules showed high α‐amylase inhibition. Sample 7 stood out for its significant inhibition against both acetylcholinesterase and butyrylcholinesterase, showcasing the diverse therapeutic potential of garlic‐derived products. Samples 3–6–7–8 demonstrated anticandidal activity against *C. albicans* and *C. tropicalis*.

## AUTHOR CONTRIBUTIONS

**Elif Ulutaş Deniz:** Conceptualization (equal); data curation (equal); formal analysis (equal); methodology (equal); writing – original draft (equal). **Hafize Yuca:** Conceptualization (equal); data curation (equal); investigation (equal); methodology (equal); writing – original draft (equal). **Bilge Aydın:** Conceptualization (equal); data curation (equal); formal analysis (equal); investigation (equal); methodology (equal); writing – original draft (equal). **Gözde Öztürk:** Conceptualization (equal); data curation (equal); formal analysis (equal); methodology (equal). **Furkan Çoban:** Conceptualization (equal); data curation (equal); investigation (equal); resources (equal); writing – original draft (equal). **Gamze Göger:** Conceptualization (equal); data curation (equal); formal analysis (equal); investigation (equal); methodology (equal); writing – original draft (equal). **Betül Demirci:** Conceptualization (equal); data curation (equal); formal analysis (equal); investigation (equal); methodology (equal); writing – original draft (equal). **Songül Karakaya:** Conceptualization (equal); data curation (equal); formal analysis (equal); investigation (equal); methodology (equal); writing – original draft (equal); writing – review and editing (equal).

## FUNDING INFORMATION

None.

## CONFLICT OF INTEREST STATEMENT

The authors have declared no conflicts of interest for this article.

## ETHICS STATEMENT

The study was approved by the Atatürk University Faculty of Pharmacy Unit Ethics Committee (Approval Number: 2400008798).

## Data Availability

The data supporting the findings of this study are available from the corresponding author upon reasonable request.
